# The COVID-19 pandemic and the Swedish strategy: Epidemiology and postmodernism

**DOI:** 10.1016/j.ssmph.2020.100643

**Published:** 2020-08-08

**Authors:** Martin Lindström

**Affiliations:** Social Medicine and Health Policy, Department of Clinical Sciences in Malmö, Lund University, S-205 02, Malmö, Sweden

**Keywords:** Pandemic, Corona virus, COVID-19, Swedish strategy, Herd immunity, Individual responsibility, Evidence-based medicine, Post-materialism, Postmodernism, Sweden

## Abstract

The aim is to outline the underlying epidemiological thinking and mentality in post-materialist and postmodern Sweden behind the Swedish strategy. The aim is not to investigate the handling of the pandemic in Sweden in the long-run. Overconfidence in herd immunity, overconfidence in individual responsibility in a pandemic needing community-centered approaches, overconfidence in evidence-based medicine and neglect to coordinate with the WHO and other countries may be associated with post-materialist values and postmodernism including opposition against modern authority, rationality and science, and also an anti-traditionalist stance towards older generations. COVID-19 epidemiology and postmodernism may be a dangerous combination.

## Introduction

The COVID-19 pandemic reached many European countries in terms of a general spread in society in late February and early March 2020, although there are indications that the contagion had been present in some individuals already in December 2019 without being known at the time. The first European country with a general spread in society was Italy. In the initially most severely affected countries Italy and Spain the gravity of the situation had initially clearly been underestimated, and this also rapidly became apparent in e.g. Belgium, France, the Netherlands and the UK. The general response throughout Europe to the outbreak of the pandemic was a lockdown of society in mid-March, with the general exception of vital societal functions, e.g. healthcare, police, fire defense, military defense, food stores, and pharmacies. These lockdowns were not identical in all European countries, but they approximately followed the recommendations regarding isolation, social distancing and mass testing from the World Health Organization (WHO), and included the closure of the entire school system, universities, skiing resorts, and often (but not always) restaurants and bars, shops, markets and other facilities judged as non-essential. Most countries closed their borders and firmly restricted meetings and social activities. Still, it seems that the lack of rapid and timely community-centered approaches, and most importantly weak public health infrastructures, may have caused a high number of infected cases and a comparatively high mortality in many European countries compared to the initially affected East Asian countries (Shokoohi et al., 2020).

In contrast to other countries, Sweden implemented a less restrictive strategy based on recommendations from the Public Health Agency (PHA) (Folkhälsomyndigheten) with great emphasis on individual responsibility. In short, the strategy aimed to protect senior and/or vulnerable citizens, and to slow down the spread of the virus so that the healthcare system would be able to cope and not collapse (Sayers, 2020). Prohibitions only included entry into the country of non-citizens from outside the EU/EEA area prohibited (17 March), all gatherings in public places of more than 500 participants (11 March), all gatherings of more than 50 participants in public places (27 March), only serving at the table allowed in restaurants and bars (24 March), and visits in homes for the elderly prohibited nationally by the government (31 March) (Obminska, 2020). In addition, visits to hospitals have been prohibited/restricted by the regions/county councils (Malmén, 2020). Recommendations included early restrictions regarding travels to some countries already early affected by the pandemic (e.g. China 26 January, Italy 6 March), restrictions regarding travel to all countries (14 March), persons with minimal symptoms recommended to stay home from work, day of qualifying period (*karensdag*) abolished, sick leave without doctor's certificate extended from 7 to 14 days (14 March), persons 70 and above should stay home and reduce social contacts, employers particularly in Stockholm should encourage work at home (16 March), and distance teaching for secondary schools (gymnasium) and universities (18 March) (Obminska, 2020).

Denmark initiated early lockdown measures. Starting on 13 March, all public sector employees in non-essential functions were ordered to stay home, private employers were urged to allow their employees to stay home and work from home if possible (with the exception of employees in e.g. vital functions and food stores and pharmacies), and all secondary schools, libraries, museums, indoor cultural institutions and similar institutions were closed. Starting on 16 March, all primary school and daycare centers were closed (Hansen, 2020). On 18 March, public assemblies of more than ten people became illegal, all stores with close contact and nightclubs were closed, and restaurants were only allowed to deliver take-way. On 23 March, it was announced that lockdown would remain until mid-April (Danmarks Radio, 2020).

On 16 March, the Finnish government declared a state of emergency. All schools were closed, except early education. Most public facilities (libraries, museums, theatres) were closed down. Essential occupations were exempted from the Working Hours and Annual Holidays Acts. A maximum 10 persons was set for public meetings. Seniors above 70 should avoid social contacts. No visitors to hospitals were allowed, except to critically ill patients. The capacity for social and health care were increased, other less critical activities decreased. Borders were shut down (Yle Uutiset, 2020). These measures were to extend to 13 April, but were later extended to 13 May.

On 12 March, the Norwegian Health Directorate introduced measures such as closure of educational institutions, discontinuation of sports activities, closure of cultural events, sports events, gyms and swimming pools, and the closure of pubs, night clubs and bars. Health care professionals were prohibited from travelling abroad until 20 April. Quarantines were introduced for everyone arriving from travels outside Finland and Sweden since 27 February. Restaurants serving food were kept open with requirements regarding social distancing. The Directorate discouraged travelling to work, by public transport and crowded places. People were requested not to visit institutions. Public transport continued in order to transport key professionals (Norwegian national broadcasting, 2020).

The long-term effects of the pandemic will only be possible to assess in the future. However, the short-term effects of the Swedish strategy in late May 2020 compared to the closest neighboring countries were apparent. On 21 May, 88 new deaths in COVID-19 were reported from Sweden to WHO, 3 in Denmark, 3 in Finland and 1 in Norway (for all countries with some calendar delay). The same day, a total 3831 deaths had been reported in Sweden, 554 in Denmark, 304 in Finland and 234 in Norway (WHO Situation Report 122, 2020). On 21 July, 20 new deaths were reported in Sweden, 0 in Denmark, 0 in Finland and 0 in Norway, with a total 5639 reported deaths in Sweden, 611 in Denmark, 328 in Finland and 255 in Norway (WHO Situation Report 183, 2020). The differences in death count between Sweden and Denmark, the two countries with the highest absolute numbers and per capita, could not simply be explained by differences in the initial spread of the contagion. On 30 March, roughly more than two weeks after the lock down in Denmark, the ratio between the death tolls in Sweden versus Denmark was 1.53 (110:72), probably approximately reflecting the spread of COVID-19 at the time of the Danish lockdown (WHO Situation Report 70, 2020), on 21 May the ratio was 6.92 (3831:554), and on 21 July 9.23 (5639:611).

The British independent sustainability rating agency Standard Ethics, based in London, lowered the ethical rating of Sweden on 21 May:

During the first phase of the COVID-19 epidemic, Swedish health policy did not comply with World Health Organization recommendations. Standard Ethics analysts believe that this produced additional risks for the Swedish and European populations. The current health policy seems to be a part of a general strategy that is not collaborative with the European Union (Standard Ethics, 2020).

The Swedish government is ultimately politically responsible for national public health policy in general as well as the Swedish strategy which is the topic of this short communication. Strategies and policies to handle public health issues are delegated to the PHA, and the government normally follows the advice from the PHA. Still, under conditions of national crisis, the government can set aside the PHA by enforcing a paragraph of the constitution (*Regeringsformen*) in order to install ministerial rule. However, the government never used this paragraph. Furthermore, the major part of the healthcare system is handled by the regions and county councils (*regioner och landsting*) within the framework of national legislation. Additionally, care of the elderly is mostly handled by the municipalities (*kommuner*), also within the framework of national legislation (Bull & Sterzel, 2019). This division of direct responsibility to meet the pandemic may be one reason for the problems of coordination of e.g. distribution of protective equipment and mass testing which required comparatively long time to be solved.

Why did the Swedish political and administrative elite adopt the strategy? Why did initially and for months a majority of the Swedish public accept it? The aim of this short communication is not to finally assess the handling of the outbreak of the pandemic by the government and the PHA, because we simply do not know how Sweden will compare in terms of death rates to other countries in the long run. Instead, the aim is to briefly outline the underlying epidemiological thinking and the mentality of post-materialist and postmodern Sweden behind the Swedish strategy.

## Methods

This short communication will identify connections between post-materialist values ad postmodern culture, and specific ideas behind the Swedish strategy which recurred in interviews and mass media coverage during the spring of 2020. The following sections will introduce post-materialism and postmodernism, present aspects of epidemiology specific to the Swedish strategy and then discuss the Swedish strategy and aspects of epidemiology in relation to postmodernism.

### Post-materialism and postmodernism

According to Ronald Inglehart's World Values Survey (WVS), Sweden has during the past decades been the most non-traditional, secularized, post-materialist and postmodern country in the world (Inglehart, 1997, 2018). The idea behind materialist as opposed to post-materialist values is built on a scarcity hypothesis and a socialization hypothesis. Materialist values which previously dominated the West concern material/economic and legal safety. The prominence of material values and law and order sprang from material scarcity and lack of sense of security, and was socialized into major parts of the population. Post-materialist values have become more common since the 1960s and are more concerned with individual freedom and human rights, because material and physical security have in recent decades not been scarce, and are thus taken for granted. According to Inglehart, this value shift started in the 1960s and 1970s in broader parts of the population initially in western countries (Inglehart, 1990).

The notion of materialist as opposed to post-materialist values is connected with the notion of modern versus postmodern culture. Modern culture entails a belief in authority, rationality, science and engineering. Modern culture repudiates tradition and religion. Postmodern culture regards authority, rationality, science and engineering as connected with the West and western materialist culture, which postmodern culture regards as problematic. Postmodern culture represents a renewed belief in tradition, which modernism repudiates, but only traditions which originate in non-western countries are esteemed by postmodernism. Postmodernism also leads to the appearance of new values and lifestyles and entails increased tolerance of ethnic, cultural, sexual and individual choices regarding how to live, based on an emphasis on individual rights. The postmodern view of science repudiates the modern view that only one objective truth exists to a particular scientific problem, even in medicine and natural sciences. The postmodern view of science also entails an emphasis on subjective personal feelings of what is true rather than the modern belief that there is one objective truth to be found following a research question (Inglehart, 1997).

### Aspects of epidemiology

A number of specific epidemiological characteristics of the initial Swedish strategy may be discerned: overconfidence in herd immunity (although officially never a part of the strategy), overconfidence in individual responsibility, overconfidence in evidence-based medicine and neglect to coordinate with other countries and the WHO.

#### Herd immunity- or just a very unclear strategy?

Herd immunity as an answer regarding how to handle the pandemic was soon dismissed by the UK government (Pallab, 2020) in the middle of March for the reason of very high expected death rates. Initial studies in the beginning of the pandemic suggested that death rates following COVID-19 infection ranging from 0.25% to 3.0% would make strategies including herd immunity unacceptable to the public (Kwok et al., 2020; Wilson et al., 2020). For example, in Sweden a 1% death rate and a herd immunity acquired with 60% of the population infected (approximately 6 million of a total 10.2 million of the population) would mean 60,000 deceased. However, the government, advised by the PHA, obviously did not see this problem or alternatively chose to ignore it. The current state epidemiologist Anders Tegnell (2013-) as well as the former state epidemiologist Johan Giesecke (1995–2005) indirectly and repeatedly communicated belief in herd immunity, although herd immunity was officially never an explicit part of the Swedish strategy. Knowledge was at that point in time even more limited than now regarding the characteristics and length of immunity after infection related to the new coronavirus; “Longitudinal serological studies are urgently needed to determine the extent and duration of immunity to SARS-CoV-2” (Kissler et al., 2020), making the idea concerning herd immunity dubious. It also became increasingly apparent, as the spring passed, that only a smaller part of the population even in Stockholm had been infected although the death number in Stockholm county already was passing a total 2000 at the end of May. The former state epidemiologist Johan Giesecke continually claimed that it was “inevitable” that everyone would eventually be infected (Grundberg Wolodarski, 2020). The current state epidemiologist, in accordance with this notion of the inevitable general spread of the contagion, repeatedly emphasized that the infection curve should be flattened in order not to put too much stress on the healthcare system. In the later part of May, it became apparent that the other Nordic countries eventually had almost no infection curve to flatten, and consequently comparatively little significant stress from the COVID-19 pandemic on the health care system to ease.

On several occasions, 22 Swedish researchers criticized what seemed to be a herd immunity strategy (Bjermer et al., 2020; Carlsson et al., 2020). The representative of the PHA replied at the official press conference after the second article that herd immunity was not a part of the Swedish strategy, thus closing all further discussion. The most critical question was almost never asked by the Swedish mass media: What was the point of very loose restrictions and holding even bars, restaurants and skiing venues open if the goal was *not* to achieve herd immunity?

#### Individual responsibility

The Swedish government and the PHA took great pride in the part of their strategy to handle the pandemic relying on individual responsibility. Much responsibility of the handling of a global pandemic has been put on the individual citizens/inhabitants of Sweden and their assumed individual sense of responsibility. Restaurants and taverns (as well as many other sectors of society) were allowed to stay open leaving much responsibility to the responsibility of the individual owners and customers to keep distance, even when affected by alcohol consumption in late evenings! In fact, in an interview with CNN, the Swedish foreign minister Ann Linde, when asked by the CNN reporter about the high death rates in Sweden, blamed Swedish winter tourists for going to Italy on skiing holiday in late February when they knew that the coronavirus had spread in northern Italy (Gorani, 2020). In the same interview, she did not mention the fact that the Swedish state epidemiologist had clearly stated before this holiday week in late February that there was no problem for the Swedish public to go skiing in northern Italy (as mentioned in the introduction, travels to Sweden from Italy were restricted on 6 March, after the holiday week). Apart from the fact that the foreign minister was simply wrong in blaming individual Swedish tourists in the CNN interview, it may be a difficult and risky preventive strategy to put major responsibility for fighting a very contagious and lethal infectious disease pandemic on individual citizens/inhabitants. At least a major part of the responsibility for fighting this societal and contextual problem should be accepted by the national government, with rapid and timely community-centered approaches and public health infrastructures (Shokoohi et al., 2020). How could a major responsibility be laid on young adult (20–29 years old) and younger middle-aged (30–39 years old) individuals to be modest with visits to restaurants and taverns and for keeping social distance during such visits in order to hinder the spreading of the contagion, when it was predominantly the old and the frail that died from the spread of the infection? This paradox of the individual responsibility part of the Swedish strategy was almost never thoroughly questioned by Swedish journalists.

#### Evidence-based medicine

When asked by interviewers about e.g. mouth protection, masks and a number of other important issues, the current state epidemiologist often answered that no evidence exists. These answers were based on the concept of evidence-based medicine. The former state epidemiologist Johan Giesecke (1995–2005) stated in an interview with CNN on 17 April:

We, or the Swedish government, decided early, in January, that the measures we should take against the pandemic should be evidence-based. And when you start looking around at the measures being taken now by different countries, you'll find that very few of them have a shred of evidence …

But we know of one that has been known for 150 years or more, that washing your hands is good for you and good for others when you're in an epidemic. But the rest, border closures, school closings, social distancing … there's almost no science behind most of this (Sayers, 2020).

Evidence-based medicine suggests that the highest level of evidence (level 1) comes from randomized controlled trials (RCTs). However, this poses a problem when a completely new infectious disease pandemic caused by a previously unknown contagion suddenly occurs. Other considerations such as results from lower level studies with other study designs, based on observational data and even practical experiences, common sense and caution rather than lack of caution not only may but should then be used. It seems from this perspective that the repeated reference to evidence-based medicine in rejecting e.g. masks and mouth protection for the public as well as early closures was misplaced.

#### Neglect to coordinate with the other Nordic countries, the EU and the WHO

Swedish governments have for decades consistently prided themselves for their and the country's international orientation. Swedish governments have also prided their country as a “humanitarian great power” internationally (Melin, 2014). However, in the case of fighting the pandemic this core value seemed to have completely vanished. There was seemingly no essential coordination with the closest neighboring countries Denmark, Finland and Norway which performed a partial but still very restrictive lock-down of many non-essential societal activities for approximately a month or more starting in mid-March, then loosing restrictions in a stepwise fashion. In contrast, the Swedish government, following advice from the PHA, kept elementary schools, restaurants, bars, as well as non-essential workplaces open. Initially, the limit for gatherings of people was 500, but this was eventually reduced to a maximum 50 persons. Also, the limit 50 persons only referred to public gatherings, not private parties, baptisms, weddings, and funerals which were only given voluntary recommendations. In particular, it seemed that crowded restaurants and bars that were very seldom closed down despite the crowding functioned as hotbeds for further spread of the contagion.

### The Swedish strategy and its relation to postmodernism

The initial Swedish response to the pandemic entailed a strategy that partly neglected the empirical observations and practical experience from East Asia. Instead, the government and the PHA implemented a strategy which indirectly implicated the attainment of herd immunity by the slow spread of the infection with COVID-19, although herd immunity was never an official part of the strategy. On 2 April, Johan Giesecke (state epidemiologist 1995–2005), contracted by the PHA as adviser, expressed the feeling that everyone else was wrong and that the Swedish strategy was right in an interview in *Dagens Industri* (Grundberg Wolodarski, 2020), despite the fact that empirical observations from East Asia pointed in another direction towards instant utilization of state and strong public health infrastructures particularly at the national level (Shokoohi et al., 2020). On 23 June, when it was apparent even from Swedish experience of the pandemic that COVID-19 is a cluster disease that does not swiftly sweep the entire population as a highly contagious influenza, he said that “the virus was not as contagious as I thought” in another interview in the same daily journal (Johansson, 2020). In accordance with the postmodern view of science, feeling of being right and everyone else being wrong overtrumped empirical observations and practical experience from East Asia where the pandemic had first occurred. The PHA seems to have constructed its own alternative truth, and this alternative truth was accepted by the government as well as the majority of the population in the most postmodern country of the world, according to Inglehart's WVS.

The strong emphasis on individual responsibility as a specific trait of the Swedish strategy is closely related the postmodern emphasis on individuality and opposition to authority. The Swedish population was almost supposed to handle a major part of the pandemic by individual action. It may be added that even the emphasis on individual responsibility, instead of extensive restrictive decisions implemented by the government in an authoritative way, stands in direct opposition to empirical observations and practical experience from East Asia (Shokoohi et al., 2020).

The emphasis on evidence-based medicine entails a major paradox. On the one hand, this emphasis in the initial phase of the pandemic in Sweden led to the rejection of e.g. total social distancing in terms of lockdown, the rejection of strong governmental authority and the effective use of public health infrastructures at the national level, the initial rejection of mass testing, the rejection of face masks, although the implementation of these measures would have been in accordance with empirical observations and practical experience. On the other hand, the emphasis on evidence-based medicine did not stop the leading decision makers from believing in their own scientific truth in the form of an assumption regarding herd immunity which was empirically questionable, assuming the swift and general spread of an assumed airborne infectious disease with characteristics similar to influenza. This paradox also seems to be in accordance with the postmodern view of science as including also alternative truths even in medicine.

The neglect to coordinate public health implementation with the WHO, the EU and the other Nordic countries seems to be partly a by-product of the postmodern view of science outlined above. Additionally, a particular sense of Swedish exceptionalism has existed in Sweden since the 1970s. The self-image of the political elite in Sweden entails the view that the Swedish government should lead the way not only in the direction of a developed welfare state but also in the postmodern direction of individual rights, progressivism, globalism, and cultural diversity. The self-image of Sweden as a “humanitarian great power” has in recent decades been adopted also by non-socialist governments (Melin, 2014). It is thus not surprising that a country with such a self-image of exceptionality would be the country in Europe most prone to choose a home-constructed strategy to handle the pandemic.

## Discussion

The Swedish strategy seems to be mostly in accordance with the postmodern view of science. The strategy included overconfidence in herd immunity (officially not a part of the strategy, but regularly advanced as an expected outcome by its proponents), overconfidence in individual responsibility, overconfidence in the highest levels of evidence-based medicine regarding protective measures (combined with the paradoxical overconfidence in the swift achievement of herd immunity and individual responsibility) and neglect to coordinate with the WHO, the EU and the other Nordic countries.

As already mentioned, postmodernism and postmodern culture opposes the modernist repudiation of tradition and religion. Paradoxically, postmodernism strongly tends to only embrace the tradition and religion of other peoples and cultures than those of the West. This may help explain the fact that postmodernism and secularism are both strong and positively correlated in Sweden in a comparison with other countries in the WVS. Criticism has been forwarded against the idolization of the current state epidemiologist and the lack of critical questions from domestic journalists regarding the strategy. The most profound critique has even concerned the almost sectarian behavior within the PHA and the mass media regarding the strategy and e.g. the repudiation of face masks without critical questions from the journalists (Majlard, 2020). In fact, in an interview in mid-May in the daily newspaper *Svenska Dagbladet* Frode Forland, the Norwegian analogue to the Swedish state epidemiologist, criticized not only the Swedish strategy but also the lack of critical questions from Swedish journalists to the PHA at the daily press conferences in the initial phase of the pandemic (Falkirk, 2020). This warrants two comments. First, critical questions from the mass media may be partly constrained by the fact that major parts of the mass media in Sweden are wholly or partly financed by the ultimately responsible political decision makers they are supposed to scrutinize. The Swedish state television and radio are 100% financed by taxes, and many other newspapers are partly financed by support from the state (*presstöd*). Second, it seems that the postmodern view of the existence of multiple truths even in medicine and natural sciences may lead to less openness and less diversity in the public discourse, not more, when the consequences of such an alternative postmodern truth become apparent.

## Conclusions

Overconfidence in herd immunity, overconfidence in individual responsibility in solving a pandemic which calls for contextual and societal solutions, overconfidence in evidence-based medicine, neglect to coordinate with the WHO and other countries, and failure to instantly react to protect its own population may be associated with post-materialist values and postmodernism including opposition against modern authority, rationality and science, and an anti-traditionalist stance towards the experience of older generations.

## Ethical statement

This article does not include empirical data or study participants.

## Author statement

I am the sole author of this manuscript.

## Declaration of competing interest

There are no conflicts of interest.
